# Association between antidepressant medication use and steroid dependency in patients with ulcerative colitis: a population-based study

**DOI:** 10.1136/bmjgast-2020-000588

**Published:** 2021-05-27

**Authors:** Jonathan Blackwell, Christopher Alexakis, Sonia Saxena, Hanna Creese, Alex Bottle, Irene Petersen, Matthew Hotopf, Richard C G Pollok

**Affiliations:** 1 Imperial College London Department of Primary Care and Public Health, London, UK; 2 Department Gastroenterology, St George's University Hospitals NHS Foundation Trust, London, UK; 3 The POP-IBD study group, London, UK; 4 Gastroenterology, Royal Surrey County Hospital NHS Foundation Trust, Guildford, UK; 5 Primary Care and Public Health, Imperial College, London, UK; 6 Dr Foster Unit, School of Public Health, Faculty of Medicine, Imperial College, London, UK; 7 Department of Primary Care & Population Health, University College London, London, UK; 8 Division of Academic Psychiatry, Institute of Psychiatry Psychology and Neuroscience, London, UK; 9 South London and Maudsley Mental Health NHS Trust, London, London, UK; 10 Institute for Infection and Immunity, St George's University of London, London, UK

**Keywords:** ulcerative colitis, brain/gut interaction, inflammatory bowel disease

## Abstract

**Background:**

Animal studies indicate a potential protective role of antidepressant medication (ADM) in models of colitis but the effect of their use in humans with ulcerative colitis (UC) remains unclear.

**Objective:**

To study the relationship between ADM use and corticosteroid dependency in UC.

**Design:**

Using the Clinical Practice Research Datalink we identified patients diagnosed with UC between 2005 and 2016. We grouped patients according to serotonin selective reuptake inhibitor (SSRI) and tricyclic antidepressant (TCA) exposure in the 3 years following diagnosis: ‘continuous users’, ‘intermittent users’ and ‘non-users’. We used logistic regression to estimate the adjusted risk of corticosteroid dependency between ADM exposure groups.

**Results:**

We identified 6373 patients with UC. Five thousand two hundred and thirty (82%) use no ADMs, 627 (10%) were intermittent SSRI users and 282 (4%) were continuous SSRI users, 246 (4%) were intermittent TCA users and 63 (1%) were continuous TCA users.

Corticosteroid dependency was more frequent in continuous SSRI and TCA users compared with non-users (19% vs 24% vs 14%, respectively, χ^2^ p=0.002). Intermittent SSRI and TCA users had similar risks of developing corticosteroid dependency to non-users (SSRI: OR 1.19, 95% CI 0.95 to 1.50, TCA: OR 1.14, 95% CI 0.78 to 1.66). Continuous users of both SSRIs and TCAs had significantly higher risks of corticosteroid dependency compared with non-users (SSRI: OR 1.62, 95% CI 1.15 to 2.27, TCA: OR 2.02, 95% CI 1.07 to 3.81).

**Conclusions:**

Continuous ADM exposure has no protective effect in routine clinical practice in UC and identifies a population of patients requiring more intensive medical therapy. ADM use is a flag for potentially worse clinical outcomes in UC.

**Summary box**

**What is already known about this subject?**

- Antidepressant medications (ADMs) are used frequently in the inflammatory bowel disease population. Animal studies have demonstrated a potential role of antidepressants to ameliorate inflammation in colitis. One study of people living with ulcerative colitis (UC) suggested steroid requirements were lower during periods of ADM use. However, other studies demonstrate comorbid anxiety and depression are associated with worse outcomes in UC. It is unclear where the balance of these factors lies with respect to ADM use in routine clinical practice in UC.

**What are the new findings?**

- This study found that continuous use of serotonin selective reuptake inhibitors (SSRIs) and tricyclic antidepressants (TCA) were both associated with a significant increase in the risk of corticosteroid dependency compared with non-users (SSRI: OR 1.62, 95% CI 1.15 to 2.27, TCA: OR 2.02, 95% CI 1.07 to 3.81).

**How might it impact on clinical practice in the foreseeable future?**

- Continuous ADM exposure in routine clinical practice in UC identifies a population of patients requiring more intensive medical therapy and is a flag for potentially worse clinical outcomes in UC.

## Introduction

Antidepressant medications (ADMs) may have a role in reducing intestinal inflammation.^1^ However, the benefits of pharmacological treatment with ADMs in patients with inflammatory bowel disease (IBD) are poorly researched. Evidence from animal models of IBD indicate that ADMs can reduce intestinal inflammation via modulation of neuro-humoral pathways.^2–5^ However, studies in humans are conflicting regarding any benefit of ADM on disease outcomes in IBD.^6–10^ A Cochrane review found no firm conclusions regarding the effects of ADMs in IBD could be drawn and further studies are required.^11^

A two-way relationship between psychological stress and disease activity in ulcerative colitis (UC) is likely.^12^ It is hypothesised that activation of the sympathetic autonomic nervous system due to stress leads to secretion of epinephrine and norepinephrine, which has been linked to activation of macrophages and mast cells, which may in turn increase bacterial adherence to gut mucosa.^1 13 14^ Furthermore, stress activates the hypothalamic pituitary adrenal axis, which can result in increased gut permeability and levels of glucocorticoids, which if prolonged may desensitise glucocorticoid receptors, paradoxically leading to a proinflammatory state.^1 15–17^ Conversely, patients with IBD have significantly higher prevalence of mood disorders and use of ADMs than matched controls,^18–21^ but it remains unclear what impact these drugs may have on disease activity when used in routine clinical practice. While these drugs may ameliorate gut inflammation through neuro-humoral pathways it is also possible their use to treat comorbid mood disorders maybe associated with poorer outcomes in IBD. It is known comorbid depression and anxiety are associated with unfavourable outcomes, not least increased mortality, for many chronic diseases including IBDs.^22–26^

In order to determine the balance of these factors, we therefore aimed to study the relationship between ADM use and disease activity, as evidenced by corticosteroid dependency as previously defined by the British Society of Gastroenterology and the European Crohn’s and Colitis organisation, using a previously characterised cohort of patients with UC from a nationally representative research database.^27–29^

## Methods

### Study design and data source

Using the Clinical Practice Research Datalink (CPRD) we undertook a study of patients diagnosed with UC in the period 2005–2016. CPRD is among the largest and best validated primary care research databases worldwide. It contains prospectively collected, patient-level, pseudonymised electronic health records derived from 674 general practices and is representative of the UK population, covering 8% of the population.^30^ General practitioners use a clinical coding system (Read codes) to record diagnoses, symptoms and prescriptions, as part of routine clinical care. Data are audited to ensure accuracy and completeness. Participating practices need to achieve and maintain ‘up to standard’ (UTS) status to continue contributing to the dataset. CPRD has been extensively validated for population-based studies of both IBD and ADM use.^31 32^

### Incident case definition

We defined incident UC cases as patients with a first ever diagnosis Read code for UC at least 1 year after registering with an ‘UTS’ practice for the period 1 January 2005 to 31 December 2016 using a published and validated methodology by Lewis *et al*.^31^ We excluded patients if they had codes for both Crohn’s disease (CD) and UC, or indeterminate codes (eg, ‘non-specific colitis’).

Patients who had a comorbid condition other than UC that might require regular or prolonged steroid use, such as patients with polymyalgia rheumatic and asthma, were also excluded to avoid potential confounding for our primary outcome measure. Patients with previous organ transplants were also excluded because of the likely use of concurrent immunosuppressant and steroid medications in this group. Patients were followed up for 3 years after the date of UC diagnosis until study end-point, de-registration, or death.

We extracted data for the most commonly prescribed tricyclic antidepressants (TCAs) and serotonin selective reuptake inhibitors (SSRIs) during the study period: amitriptyline, dosulepin, escitalopram, sertraline, citalopram, fluoxetine and paroxetine.^33^ In a preliminary analysis, we identified 170 000 ADM prescriptions among patients with IBD and calculated that the median ADM prescription length was 30 days (IQR 28–56). For the purposes of this study, we assumed that all ADM prescriptions were 30 days in length.

We categorised individuals according to TCA and SSRI use. We adapted previously validated methods to define patients who were ‘continuous users’, namely those with repeat prescriptions with a gap of no greater than 60 days between consecutive prescriptions in the first 3 years after UC diagnosis.^34^ We defined ‘intermittent users’, as patients who had at least one prescription of a TCA or SSRI in the first 3 years following UC diagnosis, but were not ‘continuous users’. Our comparator group was ‘non-users’; patients who had no prescriptions for ADMs in the first 3 years after diagnosis of UC.

### Outcome measure

We determined the proportion of patients who developed corticosteroid dependency since it is associated with poorer outcomes in patients with UC.^35^ We used the European Crohn’s and Colitis Organisation and British Society of Gastroenterology guidelines criteria to identify patients with corticosteroid dependency.^28 36^ A patient was defined as ‘corticosteroid dependent’ if they had either a prescription for corticosteroids that lasted longer than 3 months or required a repeat corticosteroid prescription within 3 months of stopping the previous corticosteroid course. We assumed that a corticosteroid course was 56 days long as British Society of Gastroenterology guidance recommends a 6–8 weeks course and a recent UK wide survey has found >98% of prescribers taper corticosteroids over 8 weeks in UC.^28^

### Covariates

We included covariates with known or likely associations with TCA and SSRI use and CS dependence. These comprised: sex, age category at UC diagnosis and era of UC diagnosis.

Younger age at diagnosis may be associated with poorer outcomes in UC including increased steroid use compared with older patient groups.^37^ Patients were subdivided into three groups denoting their age at diagnosis (<17, 17–39 and ≥40).

The introduction of new steroid-sparing treatments has resulted in changing patterns of steroid prescription for IBD.^38^ To control for this, the 12-year study period was divided into three periods of 4 years to adjust for the impact of era of UC diagnosis on our outcome measure. Era 1 covered the period from 1 January 2005 to 31 December 2008, era 2 from 1 January 2009 to 31 December 2012 and era 3 from 01 January 2013 to 31 December 2016.

### Statistical analysis

Baseline characteristics of the treatment groups were summarised using frequencies and percentages.

We compared the proportion of patients meeting the definition of corticosteroid dependency over the 3-year period in each treatment group using the χ^2^ test.

We used a logistic regression model to estimate the OR of developing corticosteroid dependency within the first 3 years of UC diagnosis given TCA and SSRI use, adjusting for sex, age at diagnosis, era of diagnosis. We adjusted the analysis for clustering effects to account for differences in prescribing patterns between contributing primary care practices.

We considered a p value of less than or equal to 0.05 to be statistically significant. All analyses were performed using STATA V.16 (Statacorp LP).

## Results

Over the study period of January 2005 to December 2016, we identified 6373 individuals with incident UC who had at least 3 years of follow-up. Five thousand two hundred and thirty (82%) had no prescriptions for either SSRIs or TCAs. Five thousand four hundred and sixty-four (85.7%) had no prescriptions for SSRIs and were classed as ‘SSRI non-users’, 627 (9.8%) were ‘intermittent SSRI users’ and 282 (4.4%) were ‘continuous SSRI users’ (table 1). Of the 6373 individuals with UC 246 (3.9%) were ‘intermittent TCA users’ and 63 (1%) were ‘continuous TCA users’ (table 2).

**Table 1:** Baseline characteristics of ulcerative colitis patients by SSRI exposure status

	SSRI non-users* n=5464	SSRI intermittent users* n=627	SSRI continuous users* n=282
Male sex	3091 (57%)	270 (43%)	128 (45%)
Age at diagnosis			
<17 years	199 (4%)	1 (0%)	0 (0%)
17–40 years	1599 (29%)	203 (33%)	82 (29%)
>40 years	3666 (67%)	419 (67%)	200 (71%)
Era of diagnosis			
2005–2008	2561 (47%)	269 (43%)	105 (37%)
2009–2012	2117 (39%)	264 (42%)	118 (42%)
2013–2016	786 (14%)	94 (15%)	59 (21%)
Smoker	431 (8%)	85 (14%)	36 (13%)
Tricyclic ADM use			
Non-users	5230 (96%)	566 (90%)	268 (95%)
Intermittent users	190 (4%)	50 (8%)	6 (2%)
Continuous users	44 (1%)	11 (2%)	8 (3%)

*Non-users defined as patients with no SSRI prescriptions in first 3 years of diagnosis.

†Intermittent users defined as patients who received at least one SSRI prescription within the first 3 years of diagnosis but were not continuous users.

‡Continuous users defined as patients with consecutive SSRI prescriptions for first 3 years after diagnosis where the interval between SSRI prescription <90 days.

ADM, antidepressant medication; SSRI, serotonin selective reuptake inhibitor.

**Table 2:** Baseline characteristics of ulcerative colitis patients by tricyclic antidepressant exposure status

	TCA non-users* n=6064	TCA intermittent users* n=246	TCA continuous users* n=63
Male sex	3351 (55%)	112 (46%)	26 (41%)
Age at diagnosis			
<17 years	197 (3%)	0 (0%)	1 (2%)
17–40 years	1778 (29%)	77 (31%)	14 (22%)
>40 years	4089 (67%)	169 (69%)	48 (76%)
Era of diagnosis			
2005–2008	2970 (46%)	112 (46%)	33 (52%)
2009–2012	2379 (39%)	98 (40%)	22 (35%)
2013–2016	895 (15%)	36 (15%)	8 (13%)
Smoker	517 (9%)	28 (11%)	7 (11%)
SSRI use			
Non-users	5230 (86%)	190 (77%)	44 (70%)
Intermittent users	566 (9%)	50 (20%)	11 (17%)
Continuous users	268 (4%)	6 (2%)	8 (13%)

*Non-users defined as patients with no TCA prescriptions in first 3 years of diagnosis.

†Intermittent users defined as patients who received at least one TCA prescriptions within the first 3 years of diagnosis but were not continuous users.

‡Continuous users defined as patients with consecutive TCA prescriptions for first 3 years after diagnosis where the interval between TCA prescription <90 days.

SSRI, serotonin selective reuptake inhibitor; TCA, tricyclic antidepressant.

Patients with ‘continuous’ and ‘intermittent’ SSRI treatment were more likely to be women and smokers compared with ‘non-users’ (table 1). 2.5% of the ‘’non-users’, 43.2% of the ‘intermittent SSRI users’ and 47.5% of ‘continuous SSRI users’ had prior SSRI treatment, respectively in the 6 months before IBD diagnosis.

### SSRIs and steroid dependency

The proportion of individuals with UC who developed corticosteroid dependency within 3 years was similar in intermittent SSRI users and non-SSRI users (14.8% vs 13.6%, p=0.38). However significantly more ‘continuous SSRI users’ developed corticosteroid dependency than non-SSRI users (18.8% vs 13.6%, p=0.01).

Intermittent SSRI use was associated with a similar adjusted risk of corticosteroid dependency compared with non-SSRI users (OR 1.19, 95% CI 0.95 to 1.50). However, continuous SSRI use was associated with a significantly higher adjusted risk of developing corticosteroid dependency (OR 1.62, 95% CI 1.15 to 2.27, table 3) compared with non-SSRI users.

**Table 3:** Logistic regression for risk of corticosteroid dependency within 3 years of diagnosis among patients with UC

	Unadjusted n=6373		Adjusted n=6373	
	OR	95% CI	OR	95% CI
Sex				
Female	1	–	1	–
Male	**1.25**	**1.09 to 1.44**	**1.29**	**1.12 to 1.48**
Age at diagnosis				
<17	**2.15**	**1.54 to 2.99**	**2.15**	**1.54 to 3.01**
17–40	1	–	1	–
>40	**0.74**	**0.63 to 0.86**	**0.72**	**0.61 to 0.84**
Era of diagnosis				
2005–2008	1	–	1	–
2009–2012	0.99	0.84 to 1.16	0.97	0.82 to 1.14
2013–2016	0.85	0.68 to 1.06	0.81	0.65 to 1.01
Smoking status				
Non-Smoker	1	–	1	–
Smoker	0.90	0.79 to 1.03	0.89	0.78 to 1.01
TCA exposure*				
Non-users	1	–	1	–
Intermittent users	1.07	0.74 to 1.55	1.14	0.78 to 1.66
Continuous users	**1.95**	**1.06 to 3.60**	**2.02**	**1.07 to 3.81**
SSRI exposure†				
Non-users	1	–	1	–
Intermittent users	1.11	0.88 to 1.39	1.19	0.95 to 1.50
Continuous users	**1.47**	**1.06 to 2.05**	**1.62**	**1.15 to 2.27**

Statistically significant results in bold.

*Non-users defined as patients with no TCA prescriptions in first 3 years of diagnosis. Intermittent users defined as patients who received at least one TCA prescriptions within the first 3 years of diagnosis but were not continuous users. Continuous users defined as patients with consecutive TCA prescriptions for first 3 years after diagnosis where the interval between TCA prescription <90 days.

†Non-users defined as patients with no SSRI prescriptions in first 3 years of diagnosis. Intermittent users defined as patients who received at least one SSRI prescriptions within the first 3 years of diagnosis but were not continuous users. Continuous users defined as patients with consecutive SSRI prescriptions for first 3 years after diagnosis where the interval between antidepressant medication prescription <90 days.

SSRI, selective serotonin reuptake inhibitor; TCA, tricyclic antidepressant; UC, ulcerative colitis.

### TCAs and steroid dependency

The proportion of individuals with UC who developed corticosteroid dependency within 3 years was similar in intermittent TCA users and non-TCA users (14.6% vs 13.8%, p=0.71). However, significantly more continuous TCA users developed corticosteroid dependency than non-TCA users (23.8% vs 13.8%, p=0.02).

Intermittent TCA use was associated with a similar adjusted risk of corticosteroid dependency compared with non-TCA users (OR 1.14, 95% CI 0.78 to 1.66). However, continuous TCA use was associated with a significantly higher adjusted risk of developing corticosteroid dependency (OR 2.02, 95% CI 1.07 to 3.81—table 3) compared with non-TCA users.

## Discussion

### Key findings

In this large nationally representative cross-sectional study of over 6000 patients with UC, we demonstrated continuous SSRI and TCA use are both associated with a significantly increased risk of corticosteroid dependency. Our findings indicate that concurrent use of ADM in routine clinical practice identifies a population of patients with UC requiring more intensive medical therapy.

### Findings in relation to other studies

We report a prevalence of ADM use of 23.1% among patients with UC in this nationally representative cohort. This is in keeping with previous studies which report a prevalence of ADM use of 24%–28%.^20 39 40^ Our findings suggest that ADM use in routine clinical practice is associated with a more severe disease course in UC, manifest as an increased risk of corticosteroid dependency.

Research investigating the impact of ADMs on outcomes in IBD is limited and reported findings are mixed and conflicting. Some studies have suggested ADM use may be associated with reduced disease activity in IBD.^41^ A study of 28 patients with UC examined steroid use in the year before and the year after ADM use.^6^ Among patients who had received an ADM—steroid prescriptions, disease relapses and endoscopic procedures were significantly less frequent in the year after treatment with ADM.

A retrospective cohort study examined clinical outcomes in patients with IBD given their exposure to ADMs.^40^ The authors found a lower adjusted incidence of initiating corticosteroids during periods of ADM exposure compared with non-exposure. The disparity with our findings is likely because we chose not to adjust for treatment with an ADM *before* UC diagnosis because of significant collinearity between this and ADM use *after* diagnosis.

To date, one randomised controlled trial has been conducted assessing the impact of ADMs on disease activity in patients with UC. Daghaghzadeh *et al* randomised 44 patients with depression and either CD or UC, to either 12 weeks of duloxetine or placebo.^8^ Those in the treatment arm reported experiencing significantly fewer symptoms, though this may have represented a reduction in functional gastrointestinal symptoms rather than reduced organic disease activity.^42 43^ A study by Sexton *et al* supports this, finding that ‘perceived stress’ is associated with IBD symptoms but not intestinal inflammation as determined by faecal calprotectin measurement.^44^

Chojnacki *et al* conducted a non-randomised trial which found a reduction in Mayo Clinic Disease Activity Index scores among individuals treated with tianeptine compared with placebo. Fewer patients in the treatment arm had a relapse of their UC than those on placebo (0/30 vs 3/30, respectively) however given the small numbers in both arms these findings should be interpreted with caution.^10^

Although there is some evidence from previous studies that both TCAs^2 4^ and SSRIs^3^ may ameliorate colitis, our findings suggest this may be outweighed by the association between a more severe UC disease process and depression.^18 45^ It is possible a bi-directional relationship between psychological comorbidity and IBD disease activity exists. A recent study by Gracie *et al* found baseline anxiety, but not depression, in quiescent IBD was associated with a subsequent increased need for steroids, and importantly also observed active IBD, as defined by a raised calprotectin, was associated with onset of anxiety, highlighting the bi-directional interaction.^46^

### Strengths and limitations

In this large population-based study, we used a validated research database representative of the general UK population making it less likely to be affected by referral centre bias. We modelled both our treatment groups and outcome on previously published work from CPRD, and our regression models included adjustments for demographic and clinical covariates.

There are limitations to the study design. CPRD contains data on all prescriptions in primary care. However, it does not capture corticosteroid prescriptions in secondary care meaning our study may have underestimated corticosteroid use in some individuals.

Steroid dependency is a surrogate marker of disease severity. However, data on mucosal inflammation, disease extent and inflammatory markers such as faecal calprotectin were not available for analysis. It is therefore possible some corticosteroids prescriptions were inappropriate, as individuals may have had functional symptoms rather than active UC. Although objective markers of disease activity were not available for analysis in this study, it is likely the clinicians prescribing corticosteroids would have had access to at least some of these. Some individuals may have developed corticosteroid dependency as a consequence of failure to escalate to thiopurine or biological therapies, which we were unable to account for in this study.^47^

We extracted prescription data for TCA and SSRI antidepressant medications. We acknowledge a small minority of patients may have been prescribed alternative ADMs not captured in this study. We did not analyse individual dose ranges of ADMs although we have previously established 83% of amitriptyline prescriptions in this population are issued at a dose of 30 mg or less per day. We also developed a model based on published research to define patients considered as having ‘intermittent’ and ‘continuous’ ADM use, allowing us to explore a dose–response relationship.^34^

We were not able to identify the specific indication for ADM prescription. However, in a cohort of primary care patients in the UK, contemporary with our study period, the indication for the majority of ADM prescriptions was either depression or anxiety.^48^ Given a significant proportion of TCA prescriptions were for low dose amitriptyline, it is likely in many cases TCAs were prescribed for other indications such as chronic pain, as higher doses are indicated for depression and anxiety. Both SSRIs and TCAs can cause gastrointestinal symptoms as side-effects, which could be interpreted as symptoms of UC, potentially resulting in inappropriate corticosteroid prescription.^49^ We also recognise the possibility that corticosteroid use itself may increase the risk of depression among patients with IBD,^50^ though their impact in this respect is usually short lived.^51^

### Implications

Our findings indicate an association between ADM use and worse clinical outcomes in UC as evidenced by increased corticosteroid dependency, underscoring the possible bi-directional relationship between psychiatric comorbidity and poorer outcomes in UC. It is well established that individuals with active UC are more likely to develop depression, but the converse may also be possible.^18^ This study reinforces the notion that management of common comorbid mental health disorders needs to be integral to the management of patients with UC.

The use of ADMs in IBD is supported by both national and international guidelines,^52 53^ although the evidence base is limited and further work to evaluate treatment approaches for depression in IBD is required.

Gastroenterologists are reportedly poor at recognising concurrent psychiatric illnesses among their patients,^54^ despite guidelines encouraging doctors to screen for these conditions.^53^ Continuous ADM use among patients with UC can be considered a surrogate marker for worse disease outcomes. Patients receiving regular ADM prescriptions should be recognised as individuals potentially at risk of a more aggressive disease course, as well as those who may require additional psychological support.

Prescribers also need to be aware patients’ adherence to medications for UC may be reduced among individuals receiving medications for depression.^55^ Further prospective studies are needed to assess the temporal relationship between mood disorders and IBD disease exacerbations. The neuro-immunomodulatory action of ADM on IBD disease activity using validated measures of inflammation is yet to be determined, although the forthcoming MODULATE trial may address the use of amitriptyline in stable UC.

## Conclusions

ADM use in routine clinical practice identifies a population of patients with UC with worse clinical outcomes manifest as increased rates of corticosteroid dependency. Clinicians should consider ADM use a flag for potentially worse clinical outcomes in the natural history of UC.

## Data Availability

Data may be obtained from a third party and are not publicly available. Data are held by a third party, namely the Clinical Practice Research Datalink, and may be obtained upon reasonable request.
